# The apoplasmic pathway via the root apex and lateral roots contributes to Cd hyperaccumulation in the hyperaccumulator *Sedum alfredii*

**DOI:** 10.1093/jxb/erw453

**Published:** 2016-12-15

**Authors:** Qi Tao, Radek Jupa, Jipeng Luo, Alexander Lux, Ján Kováč, Yue Wen, Yimei Zhou, Japenga Jan, Yongchao Liang, Tingqiang Li

**Affiliations:** 1 Ministry of Education Key Laboratory of Environmental Remediation and Ecological Health, College of Environmental and Resource Sciences, Zhejiang University, Hangzhou 310058, China; 2 Department of Plant Physiology, Faculty of Natural Sciences, Comenius University in Bratislava, Mlynska dolina B2, 842 15 Bratislava, Slovakia; 3 Department of Experimental Biology, Faculty of Science, Masaryk University, Kotlarska 2, 611 37, Brno, Czech Republic

**Keywords:** Apoplasmic bypass, cadmium, hydraulic conductance, lateral roots, root apex, *Sedum alfredii*, suberin lamellae, trisodium-8-hydroxy-1,3,6-pyrenetrisulphonic acid (PTS).

## Abstract

Although the significance of apoplasmic barriers in roots with regards to the uptake of toxic elements is generally known, the contribution of apoplasmic bypasses (ABs) to cadmium (Cd) hyperaccumulation is little understood. Here, we employed a combination of stable isotopic tracer techniques, an ABs tracer, hydraulic measurements, suberin lamellae staining, metabolic inhibitors, and antitranspirants to investigate and quantify the impact of the ABs on translocation of Cd to the xylem in roots of a hyperaccumulating (H) ecotype and a non-hyperaccumulating (NH) ecotype of *Sedum alfredii*. In the H ecotype, the Cd content in the xylem sap was proportional to hydrostatic pressure, which was attributed to pressure-driven flow via the ABs. The contribution of the ABs to Cd transportation to the xylem was dependent on the Cd concentration applied to the H ecotype (up to 37% at the highest concentration used). Cd-treated H ecotype roots showed significantly higher hydraulic conductance compared with the NH ecotype (76 vs 52 × 10^–8^ m s^–1^MPa^–1^), which is in accordance with less extensive suberization due to reduced expression of suberin-related genes. The main entry sites of apoplasmically transported Cd were localized in the root apexes and lateral roots of the H ecotype, where suberin lamellae were not well developed. These findings highlight the significance of the apoplasmic bypass in Cd hyperaccumulation in hyperaccumulating ecotypes of *S. alfredii*.

## Highlight

A portion of the cadmium taken up by the hyperaccumulator *Sedum alfredii* can be apoplasmically transported to the xylem via root apexes and lateral roots, where the suberin lamellae are not well developed.

## Introduction

Cadmium (Cd) compounds are among the most hazardous substances indexed in the priority list of the Agency for Toxic Substances and Disease Registry (ATSDR; https://www.atsdr.cdc.gov/). Environmental contamination by Cd is currently a widespread problem affecting agricultural soils. In addition to reducing the yields of staple crops, there is also a risk of human exposure to its toxic effects (Wagner, 1993; White and Brown, 2010). Cadmium-hyperaccumulating species, which are capable of accumulating and tolerating up to 100 µg Cd g^–1^ in shoots, are therefore of potential use in the development of phytoremediation approaches for Cd-contaminated soils (Zhao *et al.*, 2006; Krämer, 2010). A better understanding of the mechanisms controlling Cd influx into the root and its subsequent translocation to the shoot may lead to improvements in both genetic modifications and conventional breeding strategies to develop plants suitable for phytoremediation (McGrath and Zhao, 2003; Krämer, 2010).

In principle, Cd may traverse the root to the xylem vessels either through the interconnected cytoplasm of root cells (symplasm) or via the extracellular spaces between cells (apoplasm) (Lux *et al.*, 2011; Ying *et al.*, 2015). While numerous studies have demonstrated that Cd is symplasmically translocated with the aid of heavy metal P_1B_-ATPases (Papoyan and Kochian, 2004; Courbot *et al.*, 2007; Ueno *et al.*, 2011), much less attention has been paid to the contribution of the apoplasmic pathway. This ‘apoplasmic bypass’ in roots has been suggested as an important mechanism that facilitates transport of Ca (White, 2001; White andBroadley, 2003), Zn (White *et al.*, 2002; Baxter *et al.*, 2009), and Na (Faiyue *et al.*, 2010) to the xylem. Interestingly, a previous study (Lu *et al.*, 2010) has shown that the apoplasmic pathway contributes to transport of Ca to the xylem in a hyperaccumulating (H) ecotype of *Sedum alfredii* and, in addition, that Cd transport patterns are associated with those of Ca. Thus, by analogy with Ca, it may be expected that an apoplasmic pathway could contribute to the delivery of Cd to the xylem in H ecotypes of *S. alfredii*.

Suberin lamellae act as apoplasmic barriers that regulate or prevent non-selective uptake of ions and other solutes (Schreiber *et al.*, 1999; Soukup *et al.*, 2007). Therefore, differences in development of suberin lamellae may influence the transport of ions in roots, and especially affect the potential for the existence of an apoplasmic bypass. For example, Lux *et al.* (2004) and Vaculík *et al.* (2012) proposed that apoplasmic movement of Cd and Zn into the stele and subsequent upward translocation could vary just as a result of differences in the anatomy of the endodermis and in the development of the suberin lamellae. Furthermore, several potential entry sites allowing for an apoplasmic bypass exist in roots. The root apex, where endodermal suberin lamellae have not been fully developed, may facilitate apoplasmic solute flux (Schreiber, 2010; Lux *et al.*, 2011). In addition, the continuity of apoplasmic barriers is disrupted by the emergence of lateral roots. Lateral roots are initiated in the pericycle, extend through the cortex, and then appear on the surface of parental roots (Enstone and Peterson, 1992). Hence, ions could directly leak into the xylem of the main roots through the accidental openings present along the lateral roots (Peterson *et al.*, 1981; Ma *et al.*, 2001; Ranathunge *et al.*, 2005*a*, 2005*b*). However, the physical site of the apoplasmic bypass may not be located only in regions where lateral roots emerge, but also in the lateral roots themselves. This assumption is supported by findings of North and Nobel (1996), who observed loading of an apoplasmic tracer into the xylem of lateral roots as well as its presence in the main roots of *Opuntia ficus-indica*. To date, however, the deposition of suberin lamellae, localization of entry sites, and evaluation of their effects on Cd apoplasmic xylem loading have not been studied in roots of Cd hyperaccumulators.

*Sedum alfredii* Hance (Crassulaceae) is one of the few Cd hyperaccumulators native to China (Yang *et al.*, 2004). In this study, a hyperaccumulating (H) and a non-hyperaccumulating (NH) ecotype of *S. alfredii* were used to identify regions in the roots where Cd could penetrate to xylem apoplasmically, and to evaluate the potential contribution of the apoplasmic bypass to the markedly different Cd translocation and accumulation patterns in these two ecotypes. This study also investigated possible differences in the development of suberin lamellae and in suberin-related gene expression in the H and NH ecotypes that could be associated with their different capabilities of apoplasmic xylem loading.

## Materials and methods

### Plant culture

Seeds of the H ecotype of *S. alfredii* were collected from an old Pb/Zn mining area in Quzhou (29°17′N, 118°56′E) and seeds of the NH ecotype of *S. alfredii* were obtained from a tea plantation in Hangzhou (30°26′N, 120°20′E), Zhejiang province, China. Seeds of both ecotypes were germinated on a mixture of perlite and vermiculite moistened with deionized water. Four weeks after germination, uniform and healthy plants were selected and transferred into a half-strength Hoagland nutrient solution containing: 2.0 mM Ca^2+^, 4.0 mM NO_3_^–^, 1.6 mM K^+^, 0.5 mM Mg^2+^, 0.1 mM H_2_PO_4_^**–**^, 1.2 mM SO_4_^2**–**^, 0.1 mM Cl^**–**^, 10 µM H_3_BO_3_, 0.5 µM MnSO_4_, 5.0 µM ZnSO_4_, 0.2 µM CuSO_4_, 0.01 µM (NH_4_)_6_Mo_7_O_24_, and 20 µM Fe-EDTA. The pH of the nutrient solution was adjusted daily to 5.8 with 0.1 M NaOH or 0.1 M HCl. After 14 d of cultivation, plants were cultured in a full-strength basal solution, which was renewed every 3 d. Plants were grown in a growth chamber with a 14/10 h day/night cycle (irradiance of 400 µmol m^–2^ s^–1^), and a relative humidity of 70/85% at 26/20^o^C for 2weeks. After this period, the plants were exposed to the different experimental treatments.

### ^113^Cd influx experiment

Cadmium in a metallic form enriched by ^113^Cd (^106^Cd, 0.16%; ^108^Cd, 0.13%; ^110^Cd, 0.81%; ^111^Cd, 2.53%; ^112^Cd, 2.61%; ^113^Cd, 93.29%; ^114^Cd, 0.46%; ^116^Cd, 0.01%) was purchased from ISOFLEX (San Francisco, CA, USA) and used for determination of Cd concentrations (Li *et al.*, 2015). A total of 200 mg of the ^113^Cd was dissolved in 100 ml of 0.2 M HNO_3_. After 8weeks growth, roots of the H and NH ecotypes were submerged (six plants in each container) in 1.0 l of solution containing 2.0 mM Mes-Tris buffer (pH 5.8) and 0.5 mM CaCl_2_plus one of seven different concentrations of ^113^Cd (1.77, 4.43, 8.85, 17.70, 26.55, 35.40, and 44.25 µM). After 24 h, the treatment solution was replaced by an ice-cold desorption solution containing 2 mM Mes-Tris buffer (pH 5.8), 0.5 mM CaCl_2_, and 100 µM Cd(NO_3_)_2_ to remove most of the ^113^Cd adsorbed in cell walls (Li *et al.*, 2015). After 15min desorption, roots and shoots were harvested, weighed, and dried at 65°C for 72 h. Dry root and shoot samples were digested with HNO_3_-HClO_4_ (5:1, v/v) made up to 50 ml with distilled water, and filtered. The concentration of ^113^Cd in the digests was determined using inductively coupled plasma mass spectroscopy (ICP-MS; Agilent 7500cx, Agilent Technologies, Palo Alto, CA, USA).

### Visualization of Cd and PTS distribution in roots

A Cd-specific fluorescent dye (Leadmium^TM^ Green AM dye; Molecular Probes, Invitrogen, Calsbad, CA, USA), which can effectively detect weakly bound Cd ions, was used to investigate the spatial distribution of Cd in the plant roots (Lu *et al.*, 2008; Tao *et al.*, 2016). In addition, we used trisodium-8-hydroxy-1,3,6-pyrenetrisulphonic acid (PTS), which is frequently utilized as an effective tracer to visualize apoplasmic transport in plants (North and Nobel, 1996; Krishnamurthy *et al.*, 2014), to follow the apoplasmic transport pathway in the roots of *S. alfredii*. Using a very short-term procedure (taking less than 2 h), the concentration of PTS was set to 95.42 mM to allow detection of the tracer within the tissues (Faiyue *et al.*, 2010). Plants were placed in the basal nutrient solution and treated separately with 25 µM Cd(NO_3_)_2_or 95.42 mM PTS for 3, 6, and 9 min in the H ecotype and 30, 60, and 90 min in the NH ecotype (the different duration of treatments for the H and NH ecotypes was based on an unequal ability of the ecotypes to take up Cd and PTS apoplasmically, as found in a pre-experiment). Roots treated with Cd(NO_3_)_2_were divided into two parts. One part of the roots was cut into 60-µm thick cross-sections using a freezing microtome (MTC; SLEE medical GmbH, Mainz, Germany), stained with Leadmium^TM^ Green AM dye in the dark for 75 min, and washed three times with a buffer from the kit for 5 min. The other part of the roots was stained with the Cd probe directly and used for observation of whole-mounted roots. The variation of Cd over time was localized using a confocal laser scanning microscope (Zeiss CLSM 710; Carl Zeiss, Oberkochen, Germany) with excitation and emission wavelengths of 488 and 515 nm, respectively. Similarly, the fluorescence of PTS was detected using the CLSM, with excitation and emission wavelengths of 458 and 510 nm, respectively. The intensities of fluorescence signals of both dyes were quantified according to the method described in McCloy *et al.* (2014) using ImageJ software v. 1.50i (https://imagej.nih.gov/ij/). The data were normalized relative to the fluorescence intensity of the H and NH ecotypes treated with Cd for 3 and 30 min, respectively.

### Measurement of contribution of the apoplasmic bypass to Cd translocation

Eight-week-old plants were transferred to 100-ml glass tubes covered with aluminum foil and filled with the basal nutrient solution containing the seven different concentrations of ^113^Cd(NO_3_)_2_ (in the range of 1.77–44.25µM detailed above) enriched with 50 mg l^–1^ of PTS. The tubes were sealed with parafilm to limit weight loss by evaporation. Plants were allowed to take up PTS and ^113^Cd for 24 h, and transpiration during this period was measured by the weight difference method as described by Baxter *et al.* (2009). The treatment solution was then replaced by the basal nutrient solution to allow the transpiration stream to carry any remaining PTS and ^113^Cd to the shoot for a period of 24 h. Then, the concentrations of PTS and ^113^Cd in shoots were investigated. Leaves were harvested, weighed, and divided into two parts. Leaves used for PTS analysis were cut into fine pieces with a razor blade, transferred to vials with 10 ml of deionized water and placed in a water bath at 90°C. After 2 h, concentrations of PTS were analyzed using a Fluorescence Spectrophotometer (F-4600, HITACHI, Tokyo, Japan) at excitation and emission wavelengths of 403 and 510 nm, respectively. The other part of the leaf sample was dried, weighed, digested with HNO_3_-HClO_4_(v/v = 5:1), and used for determination of ^113^Cd concentrations using ICP-MS as described above. Concentrations of PTS and ^113^Cd in the transpiration stream (TS) were calculated as the quantity in the shoots divided by the volume of water transpired. The calculated values of the TS were normalized with respect to the external concentrations of PTS and ^113^Cd in order to calculate the transpiration stream concentration factor (TSCF) for PTS and ^113^Cd in each ecotype. The apoplasmic contribution was derived from the ratio of TSCF (PTS) to TSCF (^113^Cd).

### Measurements of Cd content in the xylem sap and the root hydraulic conductance

Measurements of xylem exudation and hydraulic conductance under hydrostatic pressure gradients were carried out according to the well-established protocols described by Miyamoto *et al.* (2001) and Krishnamurthy *et al.* (2011), but with some modifications. Eight-week-old H and NH ecotype plants were exposed to the basal nutrient solution containing 10 µM Cd(NO_3_)_2_. One week later, plants were fastened into polymethyl methacrylate chambers designed for sampling xylem exudates (see Supplementary Fig. S1 at *JXB* online). The roots were well aerated and the chambers were pressurized to 20 kPa. Then, shoots were cut off using sharp blades at ~3.0 cm above the junction of root and shoot. Immediately after cutting, the xylem sap that exuded from the cut surface within the first 2 min was discarded. Then, the sap was sampled with a syringe for exactly 20 min, collected in Eppendorf tubes, weighed, and made up to 4 ml with 5% nitric acid. The pressure in the chamber was then increased by 20 kPa and the process was repeated with stepwise increases until a pressure of 140 kPa was reached. The Cd content in the xylem sap was analyzed using ICP-MS as described above.

With the stepwise increases in pressure, the exuded volumes increased linearly with time (see Supplementary Fig. S2), suggesting a steady rate of fluid exudation. The volume flows (*J*_vr_; m^3^ m^–2^ s^–1^) were calculated from the slopes of the graphs by normalization with regards to the root surface area and plotted against the pressure applied to get *J*_vr_ curves for each treatment and ecotype. Root hydraulic conductance (*Lp*_r_) was determined from the linear region of these plots.

After measuring of the *J*_vr_ at 140 kPa, the air in the chamber was replaced with N_2_ to induce anoxia, thus allowing evaluation of the contribution of aquaporins to the total *Lp*_r_ (for details see Tournaire-Roux *et al.*, 2003 and Gloser *et al.*, 2007). When measurement of *J*_vr_ was finished, the roots were carefully spread in a thin layer of water, scanned with an automatic root scanner (STD-4800, Regent Instrument Inc., Quebec, Canada) against a contrasting background, and the root surface area was quantified with the aid of WinRHIZO software v. 2012b. (Regent Instrument Inc., Quebec, Canada; see Himmelbauer *et al.*, 2004 for details).

### Suberin deposition in the H and NH ecotypes

Eight-week old plants of the H and NH ecotypes of similar growth stage were placed into the basal nutrient solution with either 0 or 10 µM Cd(NO_3_)_2_. After 7 d, some of the roots were cut into cross-sections at regular distances away from the apex using the freezing microtome. In order to selectively stain the suberin lamellae, the sections and whole-mounted roots were submerged in a 0.01% (w/v) solution of Fluorol Yellow 088 (FY088; Sigma-Aldrich, St Louis, MO, USA) in lactic acid for 1 h at 70 °C and subsequently counterstained in an aqueous 0.1% (w/v) solution of Toluidine Blue O (Sigma-Aldrich) for 1 min (Lux *et al.*, 2005). Another set of sections was additionally stained with 0.1% Sudan Red 7B (SR 7B; Sigma-Aldrich) at room temperature for 1.5 h (Schreiber *et al.*, 2005). After staining, the samples were thoroughly washed in distilled water and observed with an epifluorescence microscope (Axioplan 2; Carl Zeiss, Oberkochen, Germany) using bright field (SR 7B) and under illumination with a UV-mercury lamp (FY088; Filter set 16).

### Real-time quantitative RT-PCR analysis

Total RNA was isolated using a RNAiso Plant mini kit (Takara Bio, Inc. Shiga, Japan) from H and NH ecotype roots treated with either 0 or 10 µM Cd(NO_3_)_2_ for 7 d. The isolated RNA was then converted to cDNA using a Primescript^TM^ RT reagent kit with gDNA eraser (Takara Bio, Inc. Shiga, Japan) according to the manufacturer’s instructions. Expressions of cytochrome P450 (*SaCYP86A1*) and 3-ketoacyl CoA synthase (*SaKCS20*) were determined by quantitative RT-PCR using SYBR Green I reagent (SYBR Premix Ex TaqII; Takara Bio, Inc. Shiga, Japan) on an Eppendorf Mastercycler Epgradient Realplex^2^ (Eppendorf AG, Hamburg, Germany) as described by Gao *et al.* (2013). The primers were designed using the *S. alfredii CYP* (*HE722454.1*) and *S. alfredii KCS* (*HE72905.1*) gene sequences obtained by transcriptome analysis of *S. alfredii* roots (Gao *et al.*, 2013). The sequences of the primers used are listed in Supplementary Table S1. Constitutively expressed *SaACTIN1*was used as an internal control. The relative quantities of the transcripts were calculated according to Pfaffl (2001).

### Measurement of Cd flux and observation of the PTS pathway in the root apex

The net Cd flux was measured non-invasively using a scanning ion-selected electrode technique (SIET; SIETsystemBIO-001A; Younger USA Sci. & Tech. Corp., MA, USA). Twelve 8-week-old H and NH ecotype plants were cultured in vessels with 1 l of the basal nutrient solution containing 25 µM Cd(NO_3_)_2_ and 95.42 mM PTS. Two hours later, the intact roots of four plants were immobilized with a dental wax (Li *et al.*, 2012) on a 6-cm Petri dish containing 10 ml of the treatment solution. The steady Cd fluxes were recorded at distances of 0–2, 2–4, 4–6, 6–8, 8–10, and >10 mm from the root apex. In each zone, the Cd fluxes were measured at seven points for 5–10 min for each point. The data obtained were converted into net Cd fluxes (pmol cm^–2^ s^–1^) using M_AGE_F_LUX_ developed by Xu-Yue Sci. & Tech. Co. Ltd (http://www.youngerusa.com/mageflux or http://xuyue.net/mageflux). Another four plants were used for detection of apoplasmic patterns in the root apexes with the aid of PTS, as described above. Roots of the remaining four plants were stained with propidium iodide (PI; Sigma-Aldrich) to delineate cell walls and observed with the CLSM at excitation and emission wavelengths of 488 nm and ≥585 nm, respectively.

### Effects of a transpiration inhibitor and a metabolic inhibitor on Cd uptake

Eight-week-old uniformly sized plants of both ecotypes were selected for the experiment. Twenty roots of similar length were chosen in each plant. The remaining roots were excised and the stem–root junction was sealed with a molten wax. A 100-ml beaker with a tap at the bottom was used as a container to control the level of a treatment solution to keep only root apexes immersed (RAI treatment) or whole roots immersed (WRI treatment). Plant roots in the beaker were immersed in the basal nutrient solution containing 25 µM Cd(NO_3_)_2_ and exposed to three different treatments: (i) control, (ii) leaves sprayed with a 2.0% transpiration inhibitor (TI; 87% paraffin-based petroleum oil and 13% surfactants), or (iii) nutrient solution enriched with 100 µM of the metabolic inhibitor carbonyl cyanide *m*-chlorophenyl hydrazone (CCCP). The apoplasmic pathway was predicted to be minimal when the plants were exposed to TI, while CCCP was used for its efficiency in inhibition of the symplasmic pathway. Plants treated with TI were re-sprayed with the inhibitor every 12 h. After 48 h, shoots of the H and NH ecotypes were collected, dried, and weighed. The concentration of Cd was analyzed by ICP-MS as described above.

### Measurement of lateral root density, anatomy, and permeability to PTS

Roots of 8-week-old H and NH ecotype plants were submerged in the basal nutrient solution containing either 0 or 10 µM of Cd(NO_3_)_2_ for 7 d. Whole-mounted roots and cross-sections of lateral roots were stained with 0.01% FY088 at 70°C for 1h. Subsequently, roots were washed with distilled water and observed with the epifluorescence microscope.

The lateral roots penetrating outward through the rhizodermis and appearing on the surface of the parent roots were divided into two types: ‘non-collar type’ (the base of lateral root lacks suberin lamellae) and ‘collar type’ (the base of lateral root has well-developed suberin lamellae; see Martinka *et al.*, 2012). The proportion of the non-collar type of lateral roots was calculated relative to the total number of penetrating lateral roots for each ecotype and treatment. For each treatment, 12–15 roots were used for the measurements.

The PTS tracer was used to check whether the discontinuity created by the emergence of lateral roots enables apoplasmic flow. Eight-week-old, intact H and NH ecotype plants were transferred to the basal nutrient solution containing 95.42 mM PTS for 2h. Roots were then washed with distilled water, mounted on a glass slide and observed as described above.

### Statistical analysis

All data were analyzed using the SPSS package (version 11.0; SPSS Inc., Chicago, IL, USA). Student’s *t*-test, one-way (ANOVA) or two-way analysis of variance (MANOVA) followed by Fisher’s LSD *post hoc* tests were performed on the datasets and the results were considered as statistically significant at *P* < 0.05. Specific statistical tests are indicated where appropriate in the text. Results are given as means ±SE, unless stated otherwise.

## Results

### Cd influx rates in roots and shoots

**Fig. 1.**
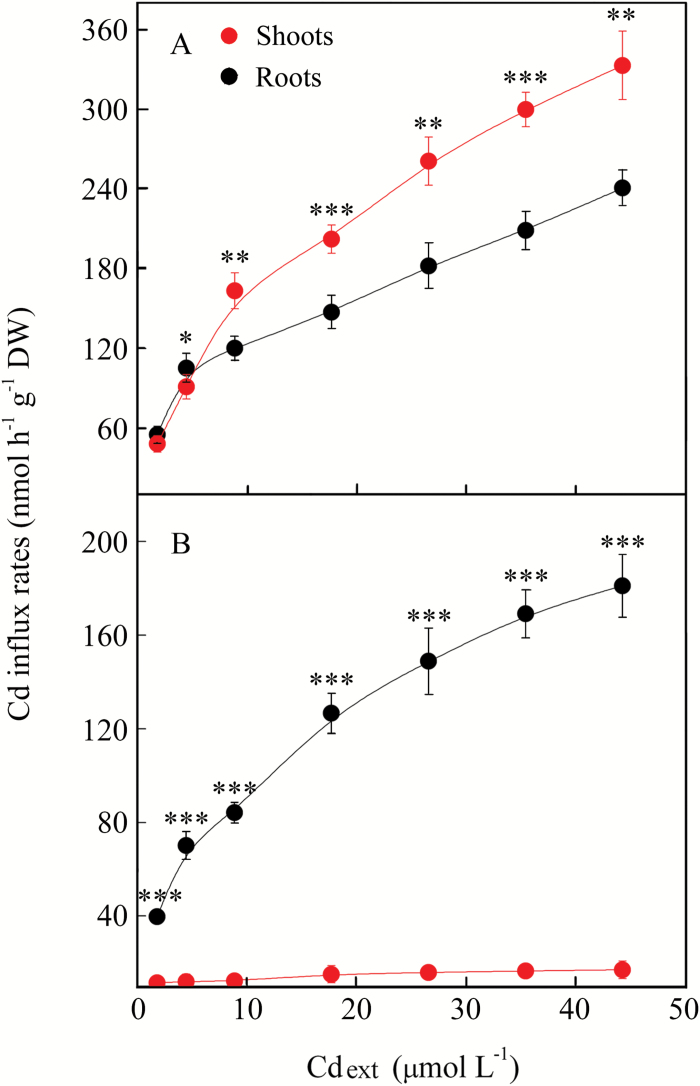
The unidirectional Cd influx rate to the roots and the shoots in intact H (A) and NH (B) plants of *Sedum alfredii* that were exposed to different external concentrations of Cd(NO_3_)_2_ (Cd_ext_). Data points and error bars represent means ±SE (*n*=3);The error bars do not extend outside some of the symbols. Asterisks indicate significant differences between root and shoot influx rates: *P<0.05, **P<0.01, and ***P<0.001 (Student’s *t*-test).(This figure is available in colour at *JXB* online.)

The kinetics of ^113^Cd influx into roots and its translocation into shoots showed different responses to Cd exposure in both the H and NH ecotypes (Fig. 1). In both the roots and shoots of the H ecotype, ^113^Cd influx rates were not saturated even at the maximum applied external concentration of ^113^Cd (Cd_ext_), which was 44.25 µM. At Cd_ext_>8.85 µM, the rate of ^113^Cd translocation to the shoots started to predominate over ^113^Cd influx into the roots (Student’s *t*-test, *P* < 0.01; Fig. 1A), and the difference increased with increasing Cd_ext_. In contrast, the rates of ^113^Cd translocation to the shoots were negligible in the NH ecotype and always significantly lower (Student’s *t*-test, *P* < 0.001) than the influx rates to the roots (Fig. 1B).

### Distribution of Cd and PTS in roots

**Fig. 2.**
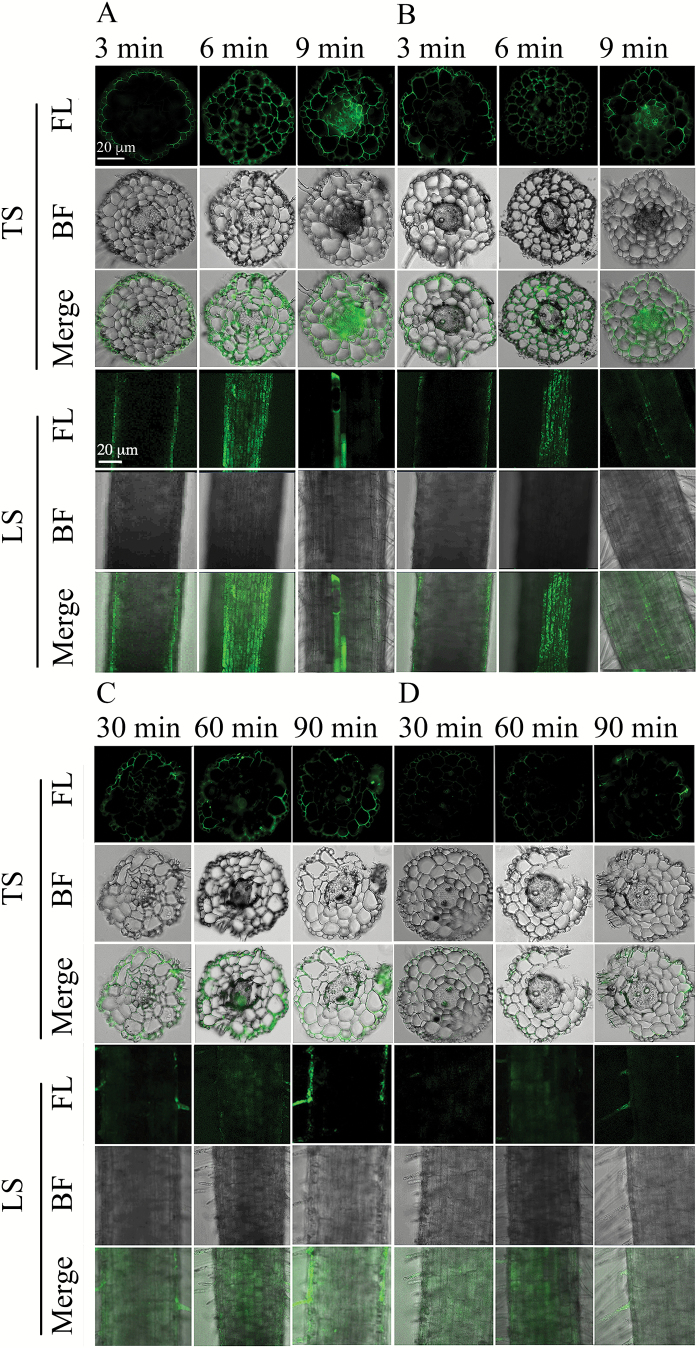
Micrographs of transverse sections (TS) and longitudinal sections (LS) of roots from H and NH ecotypes of *Sedum alfredii* exposed to 25 µM Cd(NO_3_)_2_ or 95.42 mM PTS and observed with a CLSM. The kinetics of Cd uptake were visualized using Leadmium^TM^ Green AM dye in the H ecotype (A) and the NH ecotype (C). Fluorescence of the PTS apoplasmic tracer is shown for the H ecotype (B) and the NH ecotype (D). Roots from the H ecotype were treated for 3, 6, and 9 min and roots from the NH ecotype were treated for 30, 60, and 90 min. FL, fluorescence images; BF, bright field images. (This figure is available in colour at *JXB* online.)

Staining with the fluorescent tracer Leadmium^TM^ Green AM dye revealed differences in radial penetration of Cd ions in roots of both ecotypes (Fig. 2; Supplementary Fig. S3). After exposure to 25 µM Cd for 3 min, the most intensive fluorescence appeared at the outermost layer (rhizodermis) of the H ecotype root (Fig. 2A). After exposure for 6 min, Cd moved from the rhizodermis to the cortex, and finally reached the vascular cylinder after 9 min of exposure (Fig. 2A). In line with Cd localization, the movement of PTS from the rhizodermis to the stele partially overlapped with the Cd movement (Fig. 2B). These findings were in accordance with increasing relative intensity of fluorescence of both dyes in the H ecotype within these exposure times (Supplementary Fig. S3A). In the NH ecotype, both Cd and PTS were detected in the rhizodermis and/or cortex, whereas the stele was typically free of both tracers even after 90 min of exposure (Fig. 2C, D; Supplementary Fig. S3B).

### Contribution of the apoplasmic bypass to Cd translocation to the shoots

Increases in the transpiration stream concentration factor (TSCF) of PTS and Cd corresponded with increases in Cd_ext_ in the H ecotype but not in NH ecotype, in which the values were without any apparent trend (Table 1). The ratio of the TSCF values of PTS to those of Cd in the shoots was used for estimation of the contribution of the apoplasmic bypass to the total Cd translocation to the shoots. The contribution of the apoplasmic bypass was significantly higher in the H ecotype than in NH ecotype at every Cd_ext_ concentration applied (Table 1). In the H ecotype, the contribution of the apoplasmic bypass continuously increased with Cd_ext_, from 4.34 to 36.65% (Table 1). In contrast, the apoplasmic bypass contributed only 0.20 to 3.88% with no relation to Cd_ext_ in the NH ecotype (Table 1).

**Table 1.** Concentrations of PTS and Cd in shoots and the transpiration stream, transpiration stream concentration factors (TSCF) for PTS and Cd, and the contribution of the apoplasmic bypass in hyperaccumulating (H) and non-hyperaccumulating (NH) ecotypes of *Sedum alfredii* exposed to different external concentrations of Cd. The asterisks indicate significant differences between ecotypes for a given Cd treatment: *P<0.05, **P<0.01, and ***P<0.001 (Student’s *t*-test).

Ecotype	Cd treatment (µmoll^–1^)	PTS in shoot (µgg^–1^DW)	PTS in transpiration stream (µmoll^–1^)	TSCF for PTS (×10^–3^)	Cd in shoot (µgg^–1^DW)	Cd in transpiration stream (µmoll^–1^)	TSCF for Cd (×10^–3^)	Apoplasmic bypass (%)
H	1.77	6.94	0.96	10.06	18.16	0.41	231.64	4.34**
NH		1.26	0.03	0.31	2.54	0.05	28.25	1.11
H	4.43	7.83	1.36	14.21	31.12	0.83	185.11	7.67*
NH		1.42	0.07	0. 70	3.32	0.08	18.06	3.88
H	8.85	20.12	3.36	35.20	56.02	1.85	209.04	16.84***
NH		1.63	0.08	0.84	8.81	0.22	24.89	3.37
H	17.70	32.71	5.91	61.91	136.32	5.58	315.25	19.64***
NH		1.29	0.02	0.21	13.32	0.38	21.47	0.98
H	26.55	45.85	8.25	86.49	187.91	8.42	317.38	27.25***
NH		2.11	0.05	0.05	23.34	0.56	21.09	0.24
H	35.40	53.18	8.78	92.03	245.36	10.18	287.57	32.01***
NH		1.88	0.03	0.03	18.89	0.54	15.25	0.20
H	44.25	78.64	12.91	130.52	371.30	15.76	356.16	36.65***
NH		1.23	0.02	0.02	24.71	0.41	9.27	0.22

### Cd content in xylem sap

**Fig. 3.**
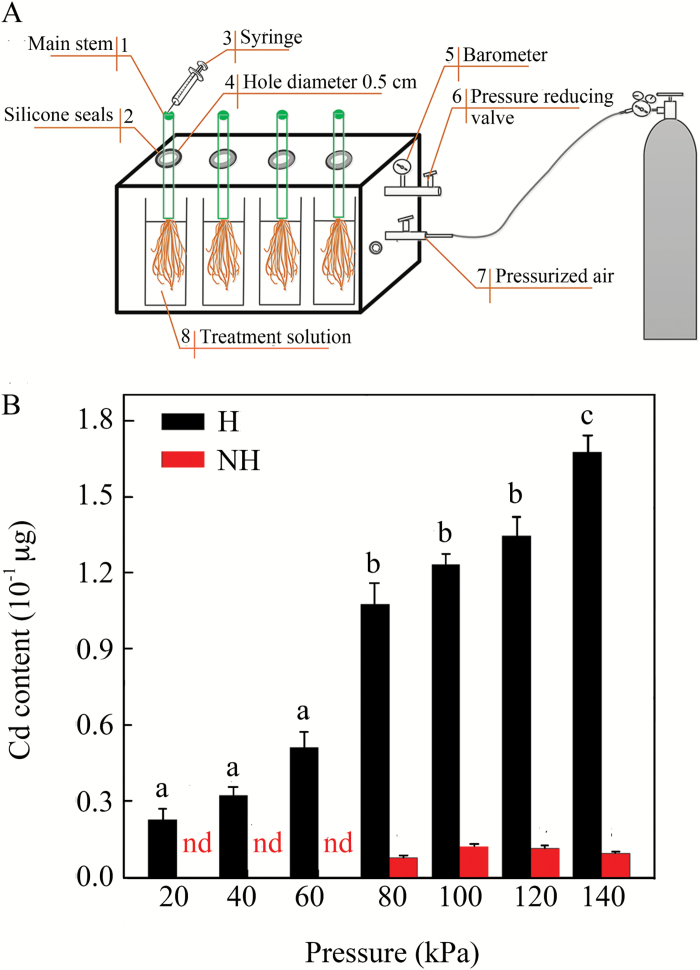
(A) Diagram of the pressure chamber designed for sampling xylem sap exudates and for measuring hydraulic conductance. (B) Cd content in the xylem sap exuded under increasing pressures for the H and NH ecotypes of *Sedum alfredii*. Data are means ±SE (*n*=3). nd, not detected. Different letters indicate significant differences within one ecotype at *P*<0.05 (one-way ANOVA). (This figure is available in colour at *JXB* online.)

Under transpiration, concentrations of both Cd and PTS in the xylem sap were significantly higher in the H than in the NH ecotype in all tested Cd_ext_ (Student’s *t*-test, *P* < 0.05; Supplementary Fig. S4). The concentration of PTS corresponded with the concentration of Cd in the xylem sap of the H ecotype, and both concentrations increased markedly with the increase in Cd_ext_ (Supplementary Fig. S4A). In the H ecotype, the Cd content in xylem sap increased with the increasing hydrostatic pressure applied to the root system with the aid of the specially designed pressure chamber (ANOVA, *F* = 23.19, *P* < 0.001; Fig. 3A, B). However, in the NH ecotype, Cd was barely detected at pressures lower than 60 kPa, and showed considerably lower contents compared to the H ecotype at any pressures used (Fig. 3B).

### Effect of Cd treatment on root hydraulic conductance

**Fig. 4.**
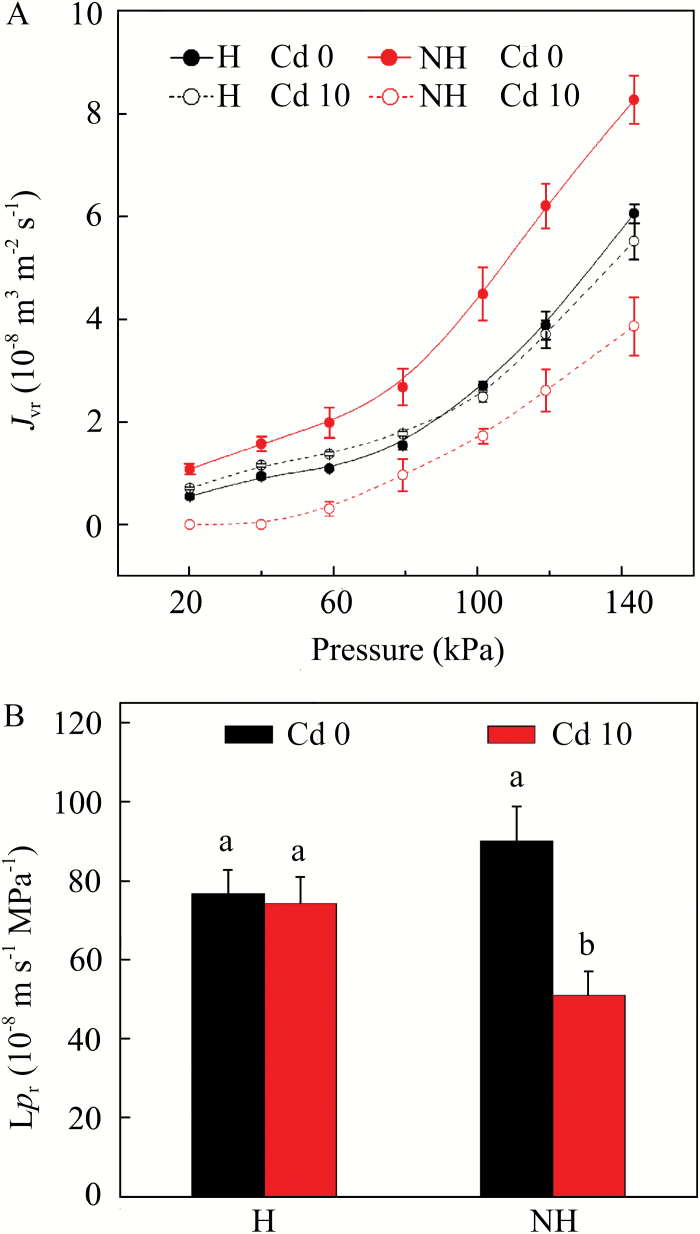
(A) Steady-state water flow of the root system normalized by surface area (*J*_vr_) plotted against applied pressure on roots of H and NH ecotypes of *Sedum alfredii* that were exposed to 0 (Cd 0) and 10 µM Cd (Cd 10) for 1week. (B) Root hydraulic conductance (*Lp*_r_) for the treatments and ecotypes as calculated from the linear slopes of the *J*_vr_ curves. Data points and error bars represent means ±SE (*n*=3). Different letters indicate significant differences at *P*<0.05 (two-way ANOVA). (This figure is available in colour at *JXB* online.)

A typical *J*_vr_ curve initially increased in a non-linear manner, suggesting the prevalence of osmotically driven uptake (Fig. 4A). At higher pressures, when the pressure-driven uptake started to predominate, the curve transformed to increase in a linear fashion. Under control conditions (no Cd), the hydraulic conductance of the root system (*Lp*_r_) was slightly higher in the NH than in the H ecotype (Fig. 4B). In response to Cd treatment, *Lp*_r_ was affected unequally in the H and NH ecotypes. After Cd treatment of 1 week, *Lp*_r_ decreased significantly in the NH ecotype (MANOVA, *F* = 11.28, *P* = 0.001), whereas Cd treatment did not bring about any substantial differences in *Lp*_r_ of the H ecotype. As a result, the *Lp*_r_ of the Cd-treated NH ecotype was significantly lower than that of H ecotype. In addition, the contribution of aquaporins to the total *Lp*_r_ was considerably higher in control NH plants than in the other three treatments (MANOVA, *F* = 19.14, *P* = 0.002; Supplementary Fig. S5).

### Development of suberin lamellae

**Fig. 5.**
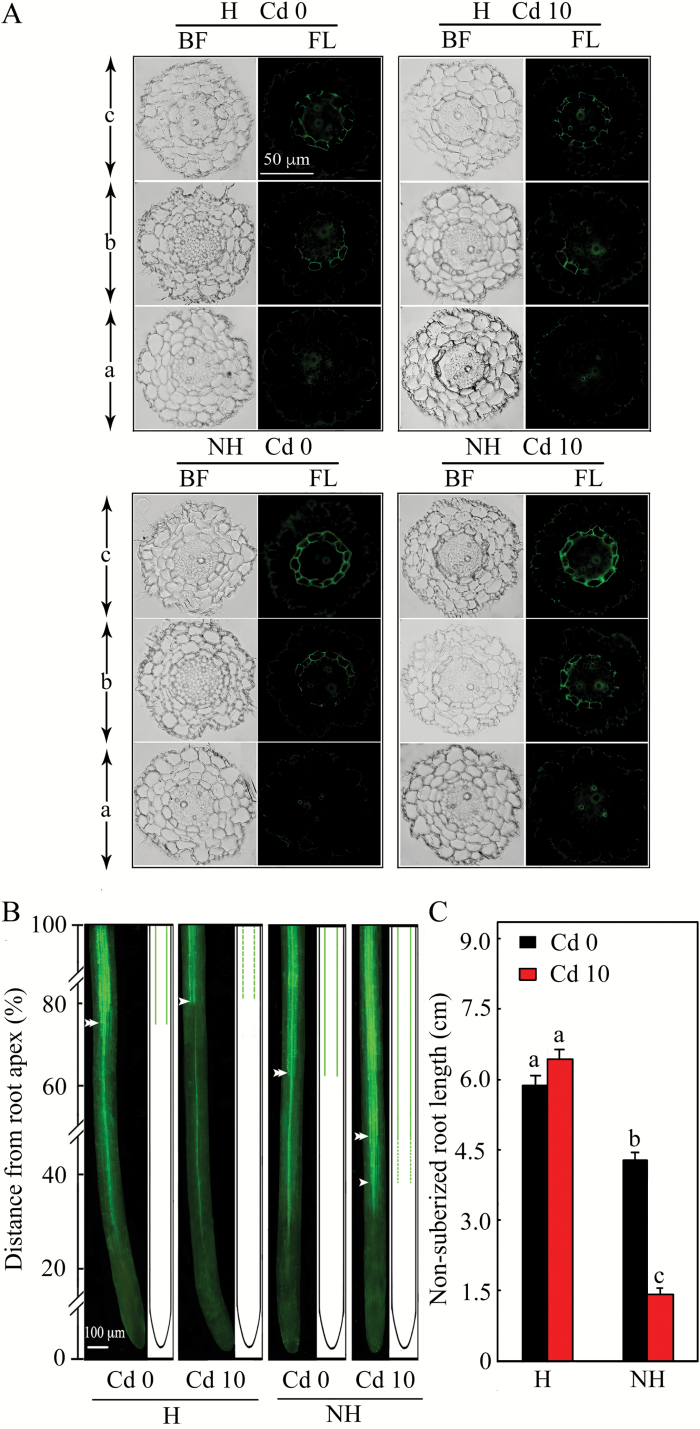
Images illustrating the development of suberin lamellae in the roots of H and NH ecotypes of *Sedum alfredii* after treatment for 7d in the basal nutrient solution containing no Cd(NO_3_)_2_ (Cd 0) or 10 µM Cd(NO_3_)_2_ (Cd 10), as observed with optical and epifluorescence microscopes. (A) In the direction from the root apex towards the base, three regions of root suberization can be distinguished after staining with Fluorol Yellow 088 and Toluidine Blue O (FY 088 + TB) on cross-sections: a region lacking suberin lamellae (*a*); a region in which the suberin lamellae are partially developed (*b*); and a region, in which suberin lamellae are fully developed (*c*). (B) Longitudinal sections of roots and accompanying diagrams showing the development of suberin lamellae. Single and double arrows represent the beginning of partially and fully developed suberin lamellae, respectively. The dotted and solid lines represent partially and fully developed suberin lamellae, respectively. (C) The length of the non-suberized part of the roots (i.e. region *a*) in the H and NH ecotypes under the Cd 0 and Cd 10 treatments. Abbreviations: BF, bright field images; FL, fluorescence images. (This figure is available in colour at *JXB* online.)

In both ecotypes, starting from the apex the roots could be divided into three regions according to the level of deposition of endodermal suberin lamellae: (*a*) not suberized, (*b*) partially suberized, and (*c*) fully suberized (Fig. 5A; Supplementary Fig. S6). An apparent difference was observed in the distance from the root apex to the beginning of regions *b* or *c* between the H and NH ecotypes. Even under control conditions, the region *c* was observed at a greater distance from the root apex in the H than in NH ecotype (Fig. 5B). After exposure to Cd, the region *b* was initiated much closer to the root apex in the NH ecotype, while slightly delayed deposition was observed in the H ecotype (Fig. 5B). Regardless of Cd treatment, the length of the non-suberized part (i.e. region *a*) was significantly greater in the H than in the NH ecotype roots (MANOVA, *F* = 115.19, *P* < 0.001; Fig. 5C).

**Fig. 6.**
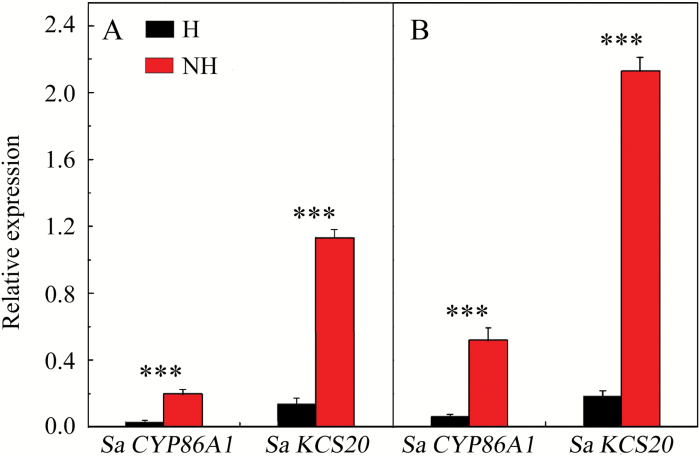
Expression analyses of *SaCYP86A1* and *SaKCS20* genes in the H and NH ecotypes of *Sedum alfredii*. Relative expression of the suberin-related genes after exposure to no Cd(NO_3_)_2_ (Cd 0) is shown in (A) and after exposure to 10 µM Cd(NO_3_)_2_ (Cd 10) is shown in (B). The expressions of *SaCYP86A1* and *SaKCS20* are shown relative to the reference *SaACTIN1*. Data represent means ±SE (*n*=3). All differences between ecotypes were statistically significant at *P*<0.001 (Student’s *t*-test). (This figure is available in colour at *JXB* online.)

Consistent with the observed differences in the development of suberin lamellae, the relative expression of *SaCYP86A1* and *SaKCS20* was significantly higher in the NH than in the H ecotype (Student’s *t*-test, *P* < 0.001; Fig. 6) regardless of treatment. Cd treatment markedly increased the expression of these two genes in the NH ecotype (Student’s *t*-test, *P* = 0.003 and *P* < 0.001 for *SaCYP86A1* and *SaKCS20*, respectively), whereas a significant increase (*P* = 0.003) was observed only for expression of *SaCYP86A1* in the H ecotype (Fig. 6).

### Cd fluxes and PTS in the root apex

**Fig. 7.**
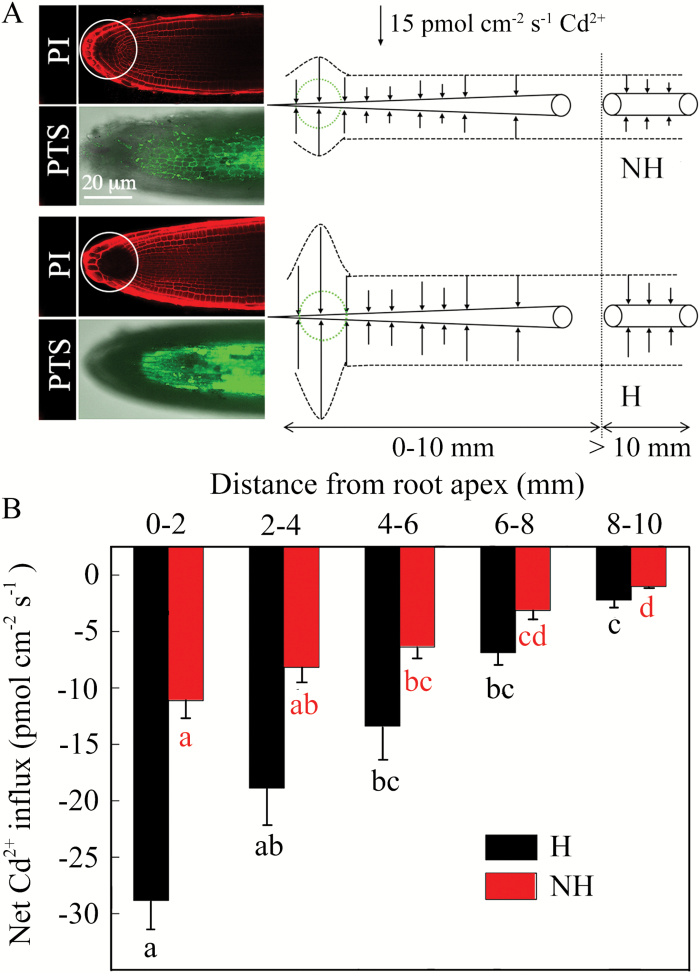
(A) Diagrams illustrating net cadmium fluxes into roots at different distances from the apexes of H and NH ecotypes of *Sedum alfredii*. The positions and magnitude of the fluxes are indicated by arrows, with arrows directed towards the root indicating influxes of Cd. The length of the arrows corresponds to the magnitude of the flux. The images on the left illustrate the fluorescence of propidium iodide (PI; red fluorescence), which delineates clear outlines of cell walls in the root apex (circled region of the columella), and the apoplasmic pathway was visualized by fluorescence of the PTS dye (green fluorescence). (B) The mean Cd fluxes in root cells of the H and NH ecotypes of *S. alfredii* at different distances from the root apex. The data are means ±SE (*n*=3). Different letters indicate significant differences within one ecotype at *P*<0.05 (one-way ANOVA). (This figure is available in colour at *JXB* online.)

After PI staining, the outlines of all cells were well delineated in the case of the NH ecotype, whereas there was a large, non-stained region in the H ecotype (white circles in Fig. 7A). In contrast, the fluorescence of PTS was more intensive in root apexes of the H ecotype compared to the NH ecotype (Fig. 7A). The results of PTS staining were consistent with the greater Cd influx observed in root apexes of the H ecotype (see diagram in Fig. 7A). Both in the H and NH ecotypes, the Cd influx was much greater in the root region close to apex (0–10 mm) than in regions at greater distance (>10 mm; Fig. 7A). We also observed a gradual decrease in net Cd influx with increasing distance from root apex in both the H and NH ecotypes (ANOVA, *F* = 7.20, *P* = 0.005 and *F* = 9.22, *P* < 0.002, respectively; Fig. 7B). Overall, the total Cd influx in the regions examined was, on average, higher in the H than the NH ecotype (Fig. 7B).

### Effect of inhibitors on symplasmic and apoplasmic Cd uptake

**Fig. 8.**
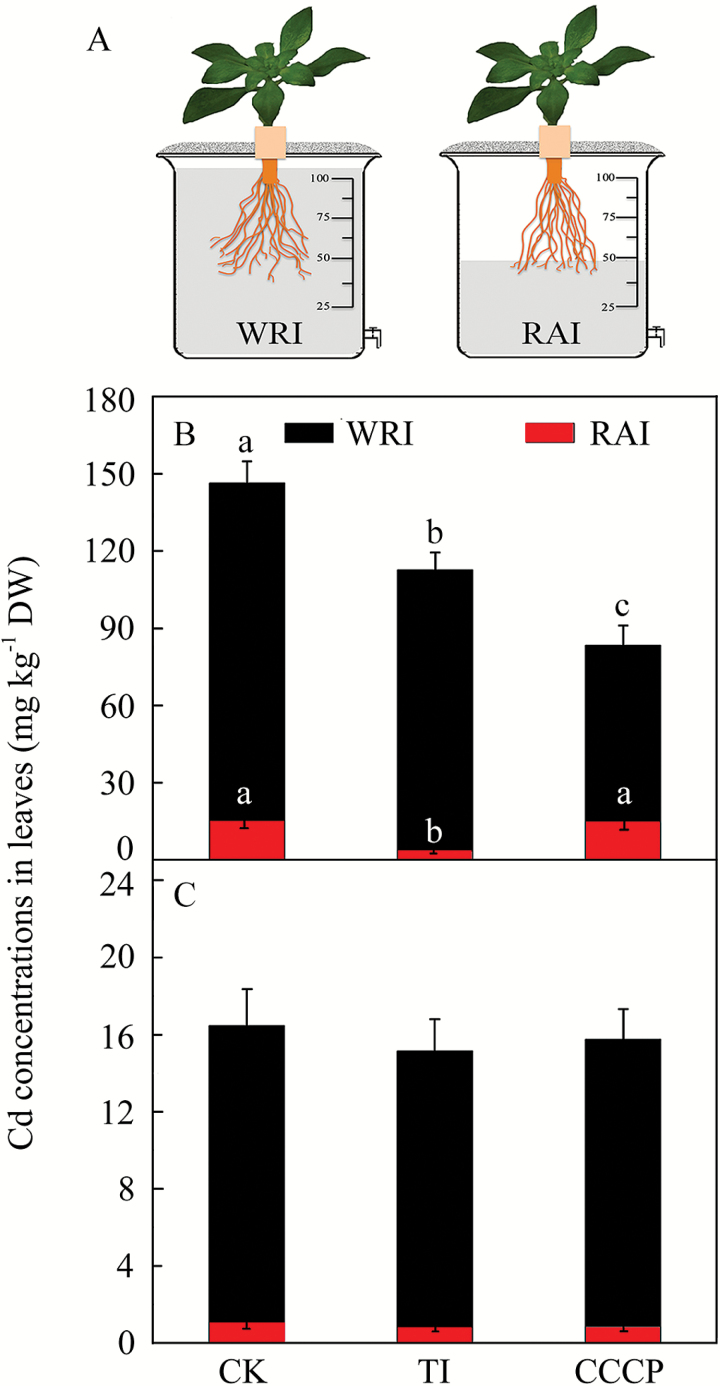
(A) Diagram of the experimental set-up for root apex immersion (RAI) and whole-root immersion (WRI) treatments. (B, C) Effects of a transpiration inhibitor (TI) and carbonyl cyanide *m*-chlorophenyl hydrazone (CCCP) on accumulation of Cd in leaves of the H ecotype (B) and the NH ecotype (C) of *Sedum alfredii*. For details about the experimental design see Methods. The data are means ±SE (*n*=3). Different letters indicate significant differences at *P*<0.05 (one-way ANOVA). (This figure is available in colour at *JXB* online.)

Root apex immersion (RAI treatment) or whole-root immersion (WRI treatment) and subsequent analysis of Cd concentration in the shoots was used to evaluate the contribution of separate root apexes for Cd influx (Fig. 8A). We found that 12.6% of the total Cd transported to the shoot was taken up by apexes in the H ecotype, while it was around 6% in the NH ecotype (Fig. 8B, C). The spraying of a transpiration inhibitor (TI) on the leaves significantly decreased the Cd concentration in the shoots of H ecotype plants receiving the RAI treatment (ANOVA, *F* = 8.84, *P* = 0.016), but only a negligible decrease was observed in the NH ecotype (Fig. 8B, C). Treatment with the metabolic inhibitor CCCP had little effect on the Cd concentration in the shoots of both H and NH ecotype plants receiving the RAI treatment (Fig. 8B, C). For the WRI treatment, application of TI caused a significant decline in Cd accumulation only in the H ecotype (ANOVA, *F* = 19.14, *P* = 0.002). A considerably greater decrease in Cd concentration was observed after application of CCCP to the H ecotype plants (Fig. 8B). In addition, under exposure to CCCP, the Cd concentration in shoots of the H ecotype increased remarkably with an increasing number of roots, while no such a steep increase was observed in the NH ecotype (Supplementary Fig. S7).

### Lateral root density, anatomy, and permeability to PTS

**Fig. 9.**
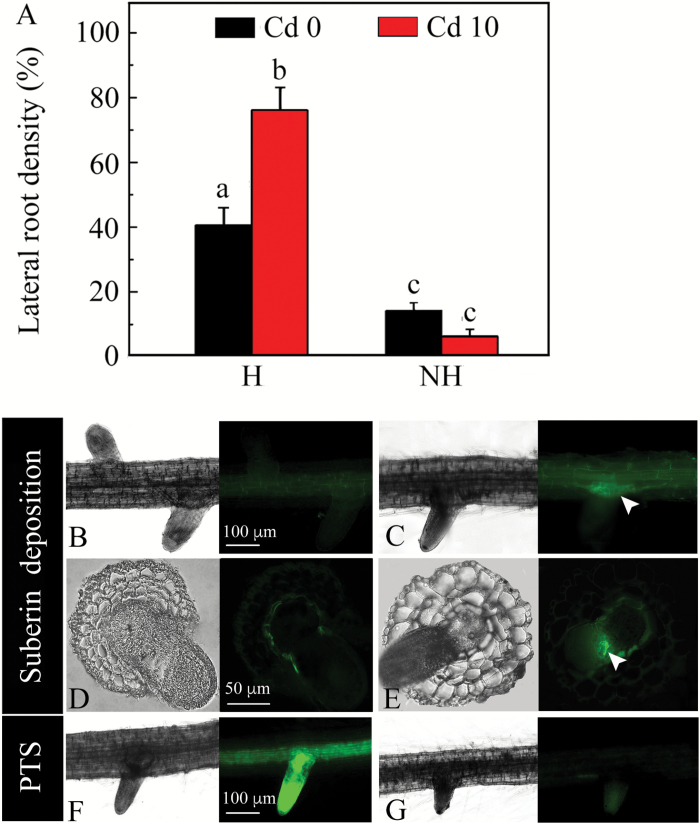
(A) Relative proportions of ‘non-collar roots’ (lateral roots without suberin lamellae in their bases) in the H and NH ecotypes of *Sedum alfredii* under exposure to no Cd (Cd 0) or 10 µM Cd(NO_3_)_2_ (Cd 10). Data represent means ±SE (*n*=12). Different letters indicate significant differences at *P* < 0.05 (two-way ANOVA). (B, C) Micrographs showing suberin lamellae deposition in the H (B) and NH (C) ecotypes of *Sedum alfredii* in longitudinal section; the arrow shows the deposition of suberin lamellae as observed with optical and epifluorescence microscopes. (D, E) Cross-sections of lateral roots of the H (D) and NH (E) ecotypes that were stained with Fluorol Yellow 088; fluorescence illustrates deposited suberin lamellae (arrow). (F, G) Longitudinal sections of a root observed with optical and epifluorescence microscopes illustrating penetration of PTS in the H (F) and NH (G) ecotypes. (This figure is available in colour at *JXB* online.)

A 7-d exposure of H ecotype roots to Cd resulted in an increase in the percentage of ‘non-collar’ lateral roots (from 40.1% to 78.3%, Fig. 9A). In contrast, a slight decrease was observed in the percentage of non-collar roots under Cd treatment in the NH ecotype. In the NH ecotype, the relative proportion of non-collar lateral roots was always significantly lower (under 20%) compared to the H ecotype, both in the presence and absence of Cd (MANOVA, *F* = 35.00, *P* < 0.001; Fig. 9A).

Almost no deposition of suberin was observed in the base of the majority of lateral roots in the H ecotype (Fig. 9B, D), as evidenced by weak fluorescence after staining with FY088 solution. In contrast, suberin lamellae were typically fully developed in the base of lateral roots in the NH ecotype, as indicated by brighter fluorescence (white arrows in Fig. 9C, E). Corresponding to the differences in deposition of suberin lamellae, PTS was detected in the stele of primary roots of the H ecotype (depicted in Fig. 9F by intensive fluorescence). No fluorescence, however, was observed in either the lateral or primary roots of NH ecotype plants (Fig. 9G).

## Discussion

A previous study showed that concentration-dependent Cd^2+^ influx in both ecotypes of *S. alfredii* yielded non-saturating kinetic curves that could be resolved into saturable and linear components (Lu *et al.*, 2008). The saturable component indicates that transport proteins on the plasma membranes probably regulate Cd influx into the symplasm, which is consistent with observations on other hyperaccumulators (Lombi *et al.*, 2001; Zhao *et al.*, 2006). However, an almost three-fold higher expression level of a Cd ion transmembrane transporter (HMA4) observed in the H ecotype is insufficient to explain the considerable differences in Cd concentration detected in the xylem sap of this species (Gao *et al.*, 2013). Therefore, the linear component of the kinetic curves, which can be interpreted as the result of a non-selective, apoplasmic Cd flux to the xylem (Lux *et al.*, 2011) and which has rarely been studied in hyperaccumulators, deserves more attention.

In the case of transport that is exclusively symplasmic, it is impossible that cation flux rates in the shoot would exceed the unidirectional cation influx to root cells (White *et al.*, 2002). However, as shown in Fig. 1, Cd translocation rates to the shoot significantly surpassed Cd influx to the root cells, supporting the hypothesis that the apoplasmic pathway may play a role in the delivery of Cd to the xylem of the H ecotype. These results are in accordance with those obtained by White *et al.* (2002), who reported that Zn could move apoplasmically to the xylem in *Thlaspi caerulescens*. Moreover, modeling of physiological and biochemical parameters by White (2001) revealed the existence of an apoplasmic bypass for Ca ions. This was later corroborated by experiments with an enhanced suberin apoplasmic barrier mutant of *Arabidopsis thaliana* (*esb1*), which showed significantly lower concentrations of Ca in its shoot compared to wild-type plants (Baxter *et al.*, 2009). In our previous study on the H ecotype of *S. alfredii*, a positive correlation was found between enhanced translocation of Cd and increased levels of exogenous Ca (Lu *et al.*, 2010). Thus, it is reasonable to speculate that Cd ions, which share some physical characteristics with Ca ions such as charge and ionic radius (Ca^2+^ = 99 pm; Cd^2+^ = 97 pm), may also move to the xylem of the H ecotype of *S. alfredii* through an apoplasmic pathway. The partial overlap of Cd and PTS pathways observed in the H ecotype (determined based on the fluorescence intensities of Leadmium Green and PTS dye; Fig. 2 and Supplementary Fig. S3A) supports this speculation and suggests that some proportion of the Cd can reach the xylem via apoplasmic bypass flow, as PTS did. However, when analyzing the fluorescence intensity of the Leadmium Green, there are a couple of general considerations that should be taken into account: (i) only weakly bound Cd ions can be detected (i.e. ions not bound to strong ligands such as phytochelatins or metalloproteins); and (ii) the fluorescence signal is not exclusively proportional to the concentration of the dye, but can be affected by interference with autofluorescence or by the pH in particular cellular compartments.

In addition to the overlapping staining, the increasing volume of exuded sap, which is proportional to the applied pressure, can also be attributed to hydrostatic pressure-driven flow via the apoplasmic bypass (Miyamoto *et al.*, 2001). In agreement with this, we determined that Cd content in the xylem sap and its exuded volume were proportional to the hydrostatic pressure applied to the H ecotype roots (Supplementary Table S2; Fig. 3). Moreover, it is likely that the relative contribution of the apoplasmic pathway to the total accumulation of toxic ions in shoots will increase with their external concentration in a nutrient solution, as has been proposed for Zn in *T. caerulescens* (White *et al.*, 2002), Na in *Oryza sativa* (Faiyue *et al.*, 2010), and Cd in *S. caprea* (Vaculík *et al.*, 2012). Consistent with these studies, our results showed that the contribution of the apoplasmic bypass was dependent on Cd concentration in the H ecotype (up to 37% at the highest concentration used), but not in the NH ecotype (Table 1). It is, however, important to emphasize that the contribution of the apoplasmic bypass to Cd translocation should be considered with caution because of two methodological limitations associated with the usage of the PTS dye as an apoplasmic tracer. First, molecules of PTS are considerably larger compared with the smaller atoms of Cd (524 vs 112 Da), suggesting their reduced potential mobility at any given structure of the apoplasmic pathway. As a consequence of the relatively lower mobility of the PTS molecules within the apoplasmic flow, the estimated contribution of the apoplasmic bypass to Cd translocation was likely to have been under-estimated to some extent in both ecotypes. Second, negatively charged cell walls interact with Cd cations and limit their mobility, whereas there is no such ionic interaction with the negatively charged molecules of PTS. In contrast to the first point, this may lead to an over-estimation of the role of the apoplasmic bypass to accumulation of Cd in shoots. Moreover, our recent findings showed that Cd bound to cell walls was retained more tightly in the NH than in the H ecotype (Li *et al.*, 2015), suggesting a more substantial over-estimation of the apoplasmic contribution in the NH ecotype. Although we have not determined the contribution of both factors to the role of the apoplasmic bypass in Cd translocation in this study, all of the aforementioned observations support our hypothesis that the apoplasmic bypass in roots contributes importantly to hyperaccumulation of Cd in *S. alfredii*. However, there is not yet evidence to explain its specific nature in terms of root anatomy and its relevance to root functioning.

Suberin lamellae belong to the effective apoplasmic barriers that separate the stele from the peripheral cell layers and thus control the apoplasmic transport of water and dissolved ions to the shoot (Reinhardt and Rost, 1995; Schreiber *et al.*, 1999; Franke and Schreiber, 2007; Schreiber, 2010). Lux *et al.* (2004) observed that differences in the development of apoplasmic barriers translate into the degree of metal tolerance and the amount of accumulation among isolates of *Salix* species. In terms of the specific changes in root anatomy, the deposition of suberin was initiated closer to the root apex in low- than in high-Cd-accumulating clones of *S. caprea* (Vaculík *et al.*, 2012). Consistent with these studies, we found that the initiation of the suberin lamellae varied significantly between the two *S. alfredii* ecotypes and also in their response to the Cd treatment (Fig. 5, Supplementary Fig. S6). Exposure to Cd induced the development of suberin lamellae closer to the root apex of the NH ecotype of *S. alfredii*, which is consistent with significantly increased expression of the two suberin-related genes (Figs 5 and 6). Similar effects have been observed in roots of other species, such as *Karwinskia humboldtiana* and *Arabidopsis thaliana* (Zelko and Lux, 2004; Martinka *et al.*, 2012). In contrast, a delayed and less extensive deposition of the suberin lamellae was observed in the H ecotype upon Cd exposure (Fig. 5).

The altered deposition of suberin lamellae translates into differences in the hydraulic conductance (*Lp*_r_) of the root system. In general, the presence of extensive apoplasmic barriers in roots reduces *Lp*_r_ (Krishnamurthy *et al.*, 2011). However, our knowledge regarding the changes in development of suberin lamellae in hyperaccumulators and their effects on water uptake and influx of toxic elements is generally very limited. Accurate quantification of *Lp*_r_ is thus crucial for assessing the xylem loading of toxic metals in particular. We found that in the absence of Cd, the NH ecotype of *S. alfredii* exhibited slightly higher *Lp*_r_ than did the H ecotype (Fig. 4), which is in contrast with the closer deposition of suberin to the root apex (Fig. 5), and also with previous results reported by Krishnamurthy *et al.* (2009, 2011). However, similar results have been reported by Ranathunge and Schreiber (2011), who found that the early development of suberin lamellae as close as 5–10 mm from the root apex in two *esb* mutants of *Arabidopsis* did not further reduce hydraulic conductivity compared with the wild-type. The slightly higher hydraulic conductivity in the NH ecotype in the absence of Cd may be attributable to an enhanced occurrence of pits in the endodermal suberin lamellae (Waduwara et al., 2008), an altered chemical composition of the suberin (Thomas *et al.*, 2007), and/or an increased expression of aquaporins in the plasma membrane and tonoplast of endodermal cells that would permit rapid transcellular flow, particularly in the root apical region (Barrieu *et al.*, 1998; Gambetta *et al.*, 2013). Aquaporins (AQPs) are water-selective channels that affect the balance in *Lp*_r_ (Aroca *et al.*, 2011; Sun *et al.*, 2016), and whose activity can be inhibited upon exposure to anoxia induced by the presence of N_2_ (Gloser *et al.*, 2007). Upon N_2_ exposure, a more pronounced decrease in *Lp*_r_ was observed in control (i.e. no Cd) NH than in control H ecotype plants (Supplementary Fig. S5), suggesting that a larger ratio of AQPs may contribute to the higher *Lp*_r_ observed in the control NH ecotype. Subjecting the NH ecotype to Cd resulted in a large reduction in *Lp*_r_ (Fig. 4), which was probably caused by a combination of altered suberin deposition (Fig. 5B) and a reduced contribution of AQPs to the total *Lp*_r_ (Supplementary Fig. S5).

Similarly to *Lp*_r_, the unequal development of apoplasmic barriers was reflected in the influx of Cd ions to roots. We observed a significant decrease in Cd influx in the basipetal direction in both the H and NH ecotypes (Fig. 7). Our findings are consistent with studies on other species, e.g. *Thlaspi caerulescens* (Piñeros *et al.*, 1998), *Triticumaestivum* (Farrell *et al.*, 2005), *Sueda salsa* (He *et al.*, 2011), *Populus canescens* (Li *et al.*, 2012), and *Helianthus annuus* (Laporte *et al.*, 2014), in which unequal Cd influx was detected with respect to distance from the root apex. It is evident that the basipetal maturation of apoplasmic barriers may limit the apoplastic route for Cd, restricting a higher influx to the root apex, where the barriers are not (or incompletely) differentiated (Lux *et al.*, 2011; Redjala *et al.*, 2011). This was confirmed by the stronger fluorescence of PTS and the most active Cd influx in regions close to the root apex of H ecotype plants in particular (Fig. 7). Similarly, application of a transpiration inhibitor combined with the root apex immersed (RAI) treatment resulted in an obvious decrease in Cd concentration only in the H ecotype (Fig. 8), which further indicates that root apexes are important regions where the apoplasmic bypass is carried out. This was indirectly supported by a relationship between the number of roots and the Cd concentration in shoots (Fig. S7), which has been observed in wheat as well (Berkelaar and Hale, 2000). With regards to the Cd translocation within the region of the root apexes, we detected a gradual decline in Cd influx into the root apical region before the suberin lamellae were deposited (compare Figs 5C and 7B). This was probably caused by the development of Casparian bands, which precedes formation of the suberin lamellae and which reduces the non-selective apoplasmic transport to some extent (Enstone *et al.*, 2002; Farrell *et al.*, 2005). Furthermore, potential decreases in the proportions of highly activated plasma membrane H^+^-ATPases (Ma *et al.*, 2014; He *et al.*, 2015) and ZIP transporters (Zinc-regulated transporter/Iron-regulated transporter-like Protein; Hart *et al.*, 1998; He *et al.*, 2009) in the direction away from the root apex may have a similar effect on the Cd influx as the development of Casparian bands.

Apart from the apexes, regions where lateral roots emerge from adventitious roots are likely to be leaky to water and solutes (Peterson *et al.*, 1981; Ma *et al.*, 2001). As there was no apoplasmic barrier in the lateral roots, Faiyue *et al.* (2010) found that the apoplasmic tracer PTS could move freely along the cortical layers of the main roots to the xylem, and be transported to the shoot. As we observed in the H ecotype of *S. alfredii*, PTS was capable of leaking directly into the xylem from the lateral roots, in which the suberin layer was not well developed (Fig. 9B, D). The results of our study suggest that the discontinuity in endodermal suberin lamellae created during the development of a lateral root from the pericycle might contribute to a direct apoplasmic penetration of ions from the external medium into the stele of the main root in the H ecotype. Similar observations have been made for other apoplasmic flow tracers such as berberine, periodic acid (HIO_4_), and ferrous ions (Fe^2+^), which were generally characterized by reaching the stele of parent roots via the vascular tissue of the lateral roots (Enstone and Peterson, 1992; Soukup *et al.*, 2002). The formation of suberin lamellae in basal cells of lateral roots of the NH ecotype (Fig. 9C, E) is similar to the collar found in lateral roots of *A. cepa* and *A. thaliana* (Peterson and Moon, 1993; Martinka *et al.*, 2012), which can restrict the entrance of PTS. The proportion of non-collar lateral roots was remarkably higher in the H than in the NH ecotype (Fig. 9A), pointing to more possibilities for apoplasmic entry. Cd treatment positively influenced the amount of non-collar lateral roots in the H ecotype (Fig. 9A), which was consistent with an enhanced contribution of the apoplasmic bypass to Cd uptake (Table 1). This suggests that the non-collar lateral roots play an important role in Cd penetration to the xylem, similarly to the apexes of main roots. Interestingly, we observed that the frequency of non-collar type lateral roots increased acropetally in the H ecotype regardless of Cd treatment (data not shown), which may substantially contribute to Cd uptake predominating in the root apical region of this ecotype.

In conclusion, the data in this study demonstrate that a part of the Cd was translocated into the xylem via the apoplasmic pathway. The results also indicate that differences in the apoplasmic contribution caused by contrasting deposition of suberin lamellae and expression of suberin-related genes lead to ecotypic differences in Cd accumulation in *S. alfredii*. The relationship between the development of the apoplasmic barrier and the hydraulic conductance of roots under exposure to Cd provide some new insights to the importance of the apoplasmic bypass to Cd hyperaccumulation. To our knowledge, this is the first report that reveals the entry sites and assesses the relative contribution of the apoplasmic pathway to Cd uptake in hyperaccumulators in detail.

## Supplementary Material

Supplementary Data
