# Supplementary material for: The apoplasmic pathway via the root apex and lateral roots contributes to Cd hyperaccumulation in the hyperaccumulator Sedum alfredii
Source: J Exp Bot. 2016 Dec 15;68(3):739–51. doi: 10.1093/jxb/erw453 (PMC5441904; doi:10.1093/jxb/erw453)

The apoplasmic pathway via the root apex and lateral roots contributes to Cd hyperaccumulation in the hyperaccumulator *Sedum alfredii*

Qi Tao, Radek Jupa, Jipeng Luo, Alexander Lux, Ján Kováč, Yue Wen, Yimei Zhou, Japenga Jan, Yongchao Liang, and Tingqiang Li

SUPPLEMENTARY DATA

Table S1. Gene-specific primers used for qRT-PCR

Genes	Forward primer	Reverse primer
SaCYP86A1	TACGGCCGATCCGAAGAATC	CCACTTGTCACCATCCGAGT
SaKCS20	CCTCCGTGTAAGCCAACTCA	ATACATGCTGGAGGACGAGC
SaACTIN1	TGTGCTTTCCCTCTATGCC	CGCTCAGCAGTGGTTGTG

Table S2. The volumes of xylem sap sampled within 20 min under increasing pressure, and concentrations of Cd in the sap in H and NH ecotype of *S. alfredii*.

Pressure	Time	H		NH	
		Volume	Cd concentration	Volume	Cd concentration
kPa	min	ml	μg ml^{-1}	ml	μg ml^{-1}
20		0.0308	0.7361	nd	nd
40		0.0551	0.5859	nd	nd
60		0.09	0.5685	0.0013	nd
80	20	0.1072	1.0029	0.0095	0.8154
100		0.1375	0.8961	0.0301	0.4035
120		0.1937	0.6949	0.0517	0.2187
140		0.2889	0.5800	0.0714	0.1324

Fig. S1. Practical experimental pressure chamber with the barometer showing real-time pressure inside (60, 80 and 100 kPa).

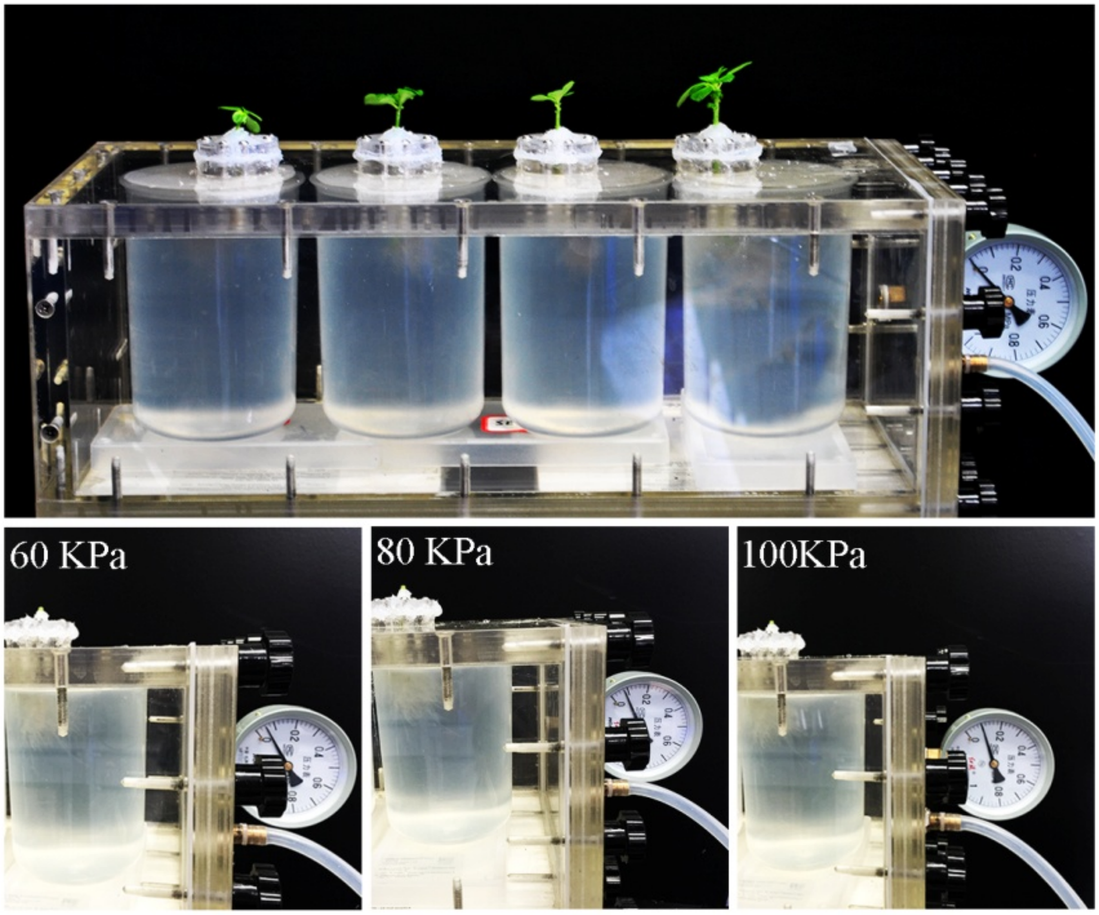

Fig. S2. Volume of exuded xylem sap as a function of time measured at different pressures in *Sedum alfredii*. Eight-week old H and NH plants were exposed to the nutrient solution containing no cadmium (Cd 0) or 10 µM $Cd(NO_3)_2$ (Cd 10) for 7 d. (A) H Cd 0, (B) NH Cd 0, (C) H Cd 10, (D) NH Cd 10.

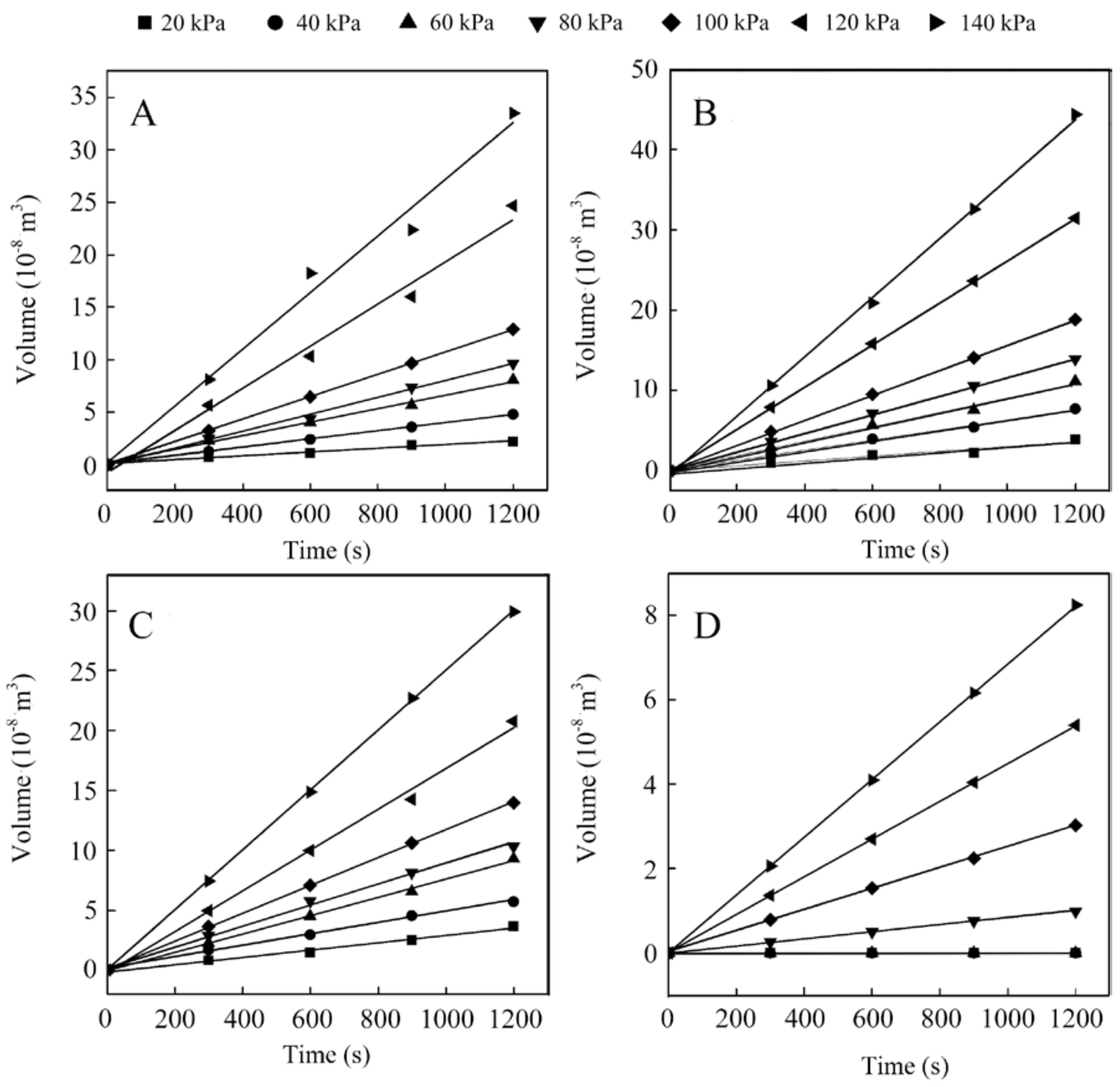

Fig. S3. The relative intensity of fluorescence measured in cross sections of roots of H (A) and NH (B) ecotype of *Sedum alfredii* at three different times and treated with Cd (black bars) or PTS fluorescent dye (red bars). To visualize presence of Cd, the sections were stained with Leadmium^{TM} dye and the fluorescence intensity of both dyes was measured with ImageJ software. The data were normalized relative to mean fluorescence intensity measured for the Cd treatment in time 3 and 30 min, respectively. Letters indicate significant differences at $P < 0.05$ (one-way ANOVA). Results are in the form mean ± SE (n=3). Note the different period of exposure between both ecotypes.

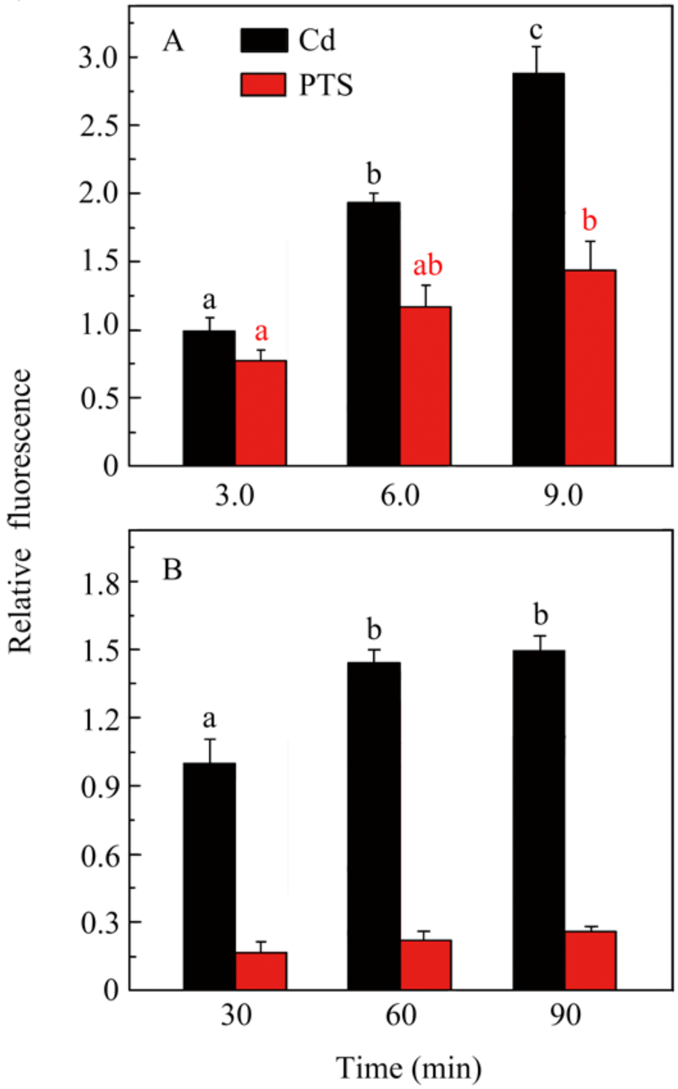

Fig. S4. The concentration of Cd (red circles) and PTS (black circles) in xylem sap of H (A) and NH (B) ecotype of *Sedum alfredii* under different external Cd (Cd_{ext}) exposure. Data are means ± SE (n=3). Error bars do not extend outside of some symbols.

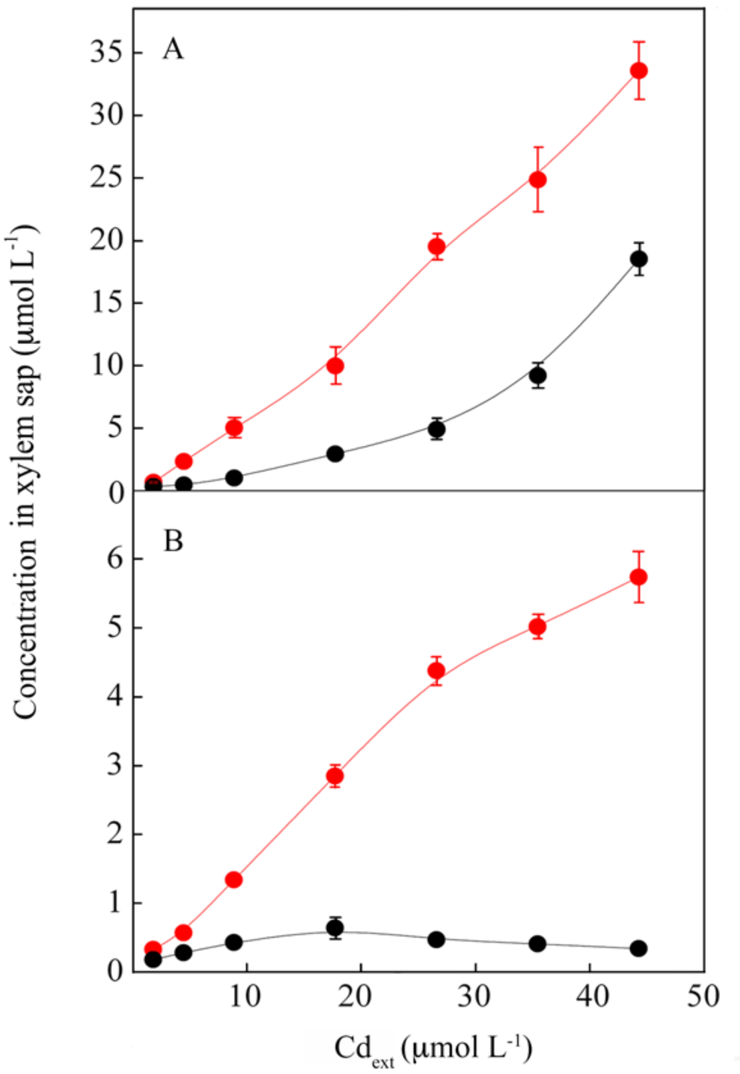

Fig. S5. (A) Hydraulic conductance of root system of H and NH ecotype of *Sedum alfredii* measured in well-aerated conditions (O_2) and under anoxia (N_2). The plants were exposed to the nutrient solution containing no cadmium (Cd 0) or 10 µM Cd (Cd 10) for 7 days. One and two asterisks indicate differences in conductance at level of significance $P < 0.05$ and $P < 0.01$, respectively (Student's *t*-test). (B) Aquaporin ratio (AR) was calculated as: AR= $[Lp_r(O_2) - Lp_r(N_2)] / Lp_r(O_2)$. Data are means ± SE (n=3). Letters indicate significant differences at $P < 0.05$ (two-way ANOVA).

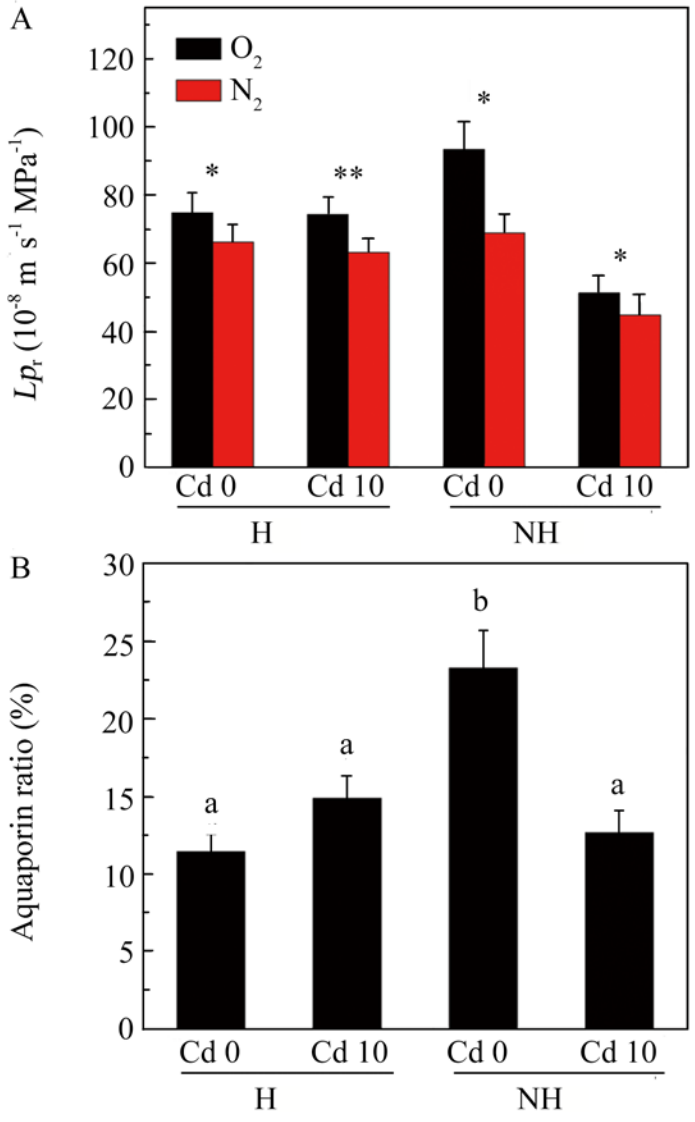

Fig S6. Cross sections showing development of suberin lamellae (indicated by white arrows), as observed with optical and epifluorescence microscope. The sections were stained with Fluorol yellow 088 and counterstained with Toluidine Blue O (FY 0888 + TB) as well as with Sudan red 7B (SR 7B). In direction from root apex to base, three regions of the root suberization can be distinguished: a region lacking suberin lamellae (*a*); a region, in which the suberin lamellae are partially developed (*b*); a region, in which suberin lamellae are fully developed (*c*).

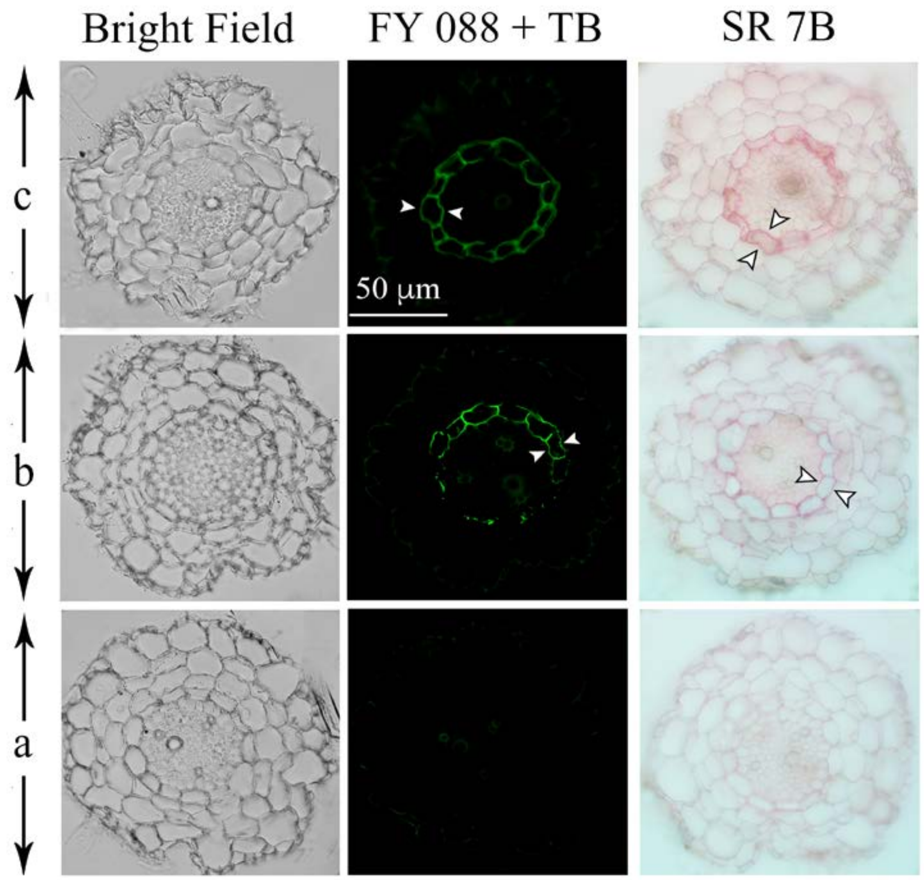

Fig. S7. Relationship between root number and Cd concentration in shoots of H (red circles) and NH (black circles) ecotype of *Sedum alfredii* under cyanide *m*-chlorophenyl hydrazone (CCCP) exposure. Data are means ± SE (n=3). Error bars do not extend outside of some symbols.

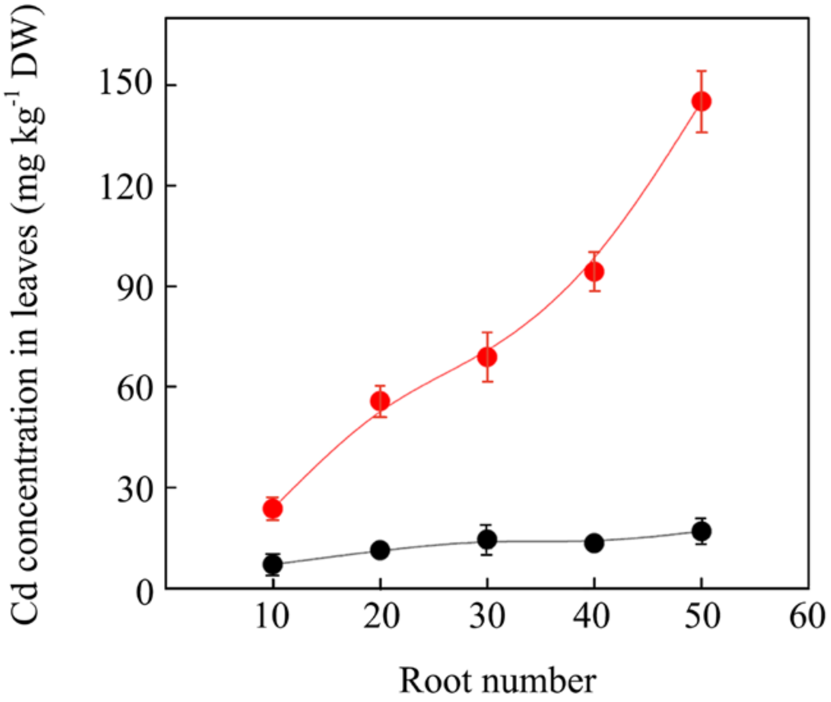

Supplement: Supplementary Data [file erw453_Supplementary_Data.zip › supplementary_tables_S1_S2_figures_S1_S7.pdf]
